# Consensus-building around the conceptualisation and implementation of sustainable healthy diets: a foundation for policymakers

**DOI:** 10.1186/s12889-022-13756-y

**Published:** 2022-08-04

**Authors:** Anna Bach-Faig, Kremlin Wickramasinghe, Natalia Panadero, Sergi Fàbregues, Holly Rippin, Afton Halloran, Ujué Fresán, Mary Pattison, João Breda

**Affiliations:** 1 FoodLab Research Group (2017SGR 83), Faculty of Health Sciences, Universitat Oberta de Catalunya (UOC), 08018 Barcelona, Spain; 2 WHO European Office for the Prevention and Control of Noncommunicable Diseases, Moscow, 125009 Russia; 3 Faculty of Health Sciences, Universitat Oberta de Catalunya (UOC), 08018 Barcelona, Spain; 4 Department of Psychology and Education, Universitat Oberta de Catalunya (UOC), 08018 Barcelona, Spain; 5 e-Health Group, Instituto de Salud Global Barcelona (ISGlobal), 08003 Barcelona, Spain; 6 Athens Office Quality of Care, Division of Country Health Policies and Systems, WHO Greece, 10675 Athens, Greece

**Keywords:** Diet, Food, Sustainability, Environment, Planetary Health, Qualitative Research, Food Policy

## Abstract

**Background:**

Healthy and sustainable diets need to be adopted to reduce the negative impact of food consumption on human and planetary health. Food systems account for a third of greenhouse gas emissions. “Dietary Patterns for Health and Sustainability” is a World Health Organization (WHO) project that aims to build consensus among international food, health, and sustainability experts and policymakers on how to conceptualise healthy and sustainable diets and on the actions and policies that could be implemented in the WHO European Region to promote these diets.

**Methods:**

A qualitative study among European food, health, and sustainability experts and policymakers to elicit their views on multiple dimensions of food sustainability and health was carried out using a three-phase process, including semi-structured interviews, a Nominal Group Technique, and focus groups during a participatory WHO workshop held in Copenhagen. Thematic analysis was used to analyse the three data sources.

**Results:**

The workshop resulted in a shared understanding of the interconnected components of sustainable healthy eating habits. As a result of this understanding, a variety of potential solutions were identified, including actions across different policy domains, tools, strategic guidelines, needs, and pathways for sustainable healthy diets. The pathways included the need for a multi-stakeholder approach, as well as the simultaneous execution of an aligned and coherent mix of policies at the local and national levels.

**Conclusions:**

The prioritised actions should be aimed at helping government policymakers promote sustainable healthy diets and make decisions on improving dietary patterns for citizens’ health and wellbeing in line with the United Nations Sustainable Development Goals in the European Region.

**Supplementary Information:**

The online version contains supplementary material available at 10.1186/s12889-022-13756-y.

## Background

The notion of planetary health implies that the health of the global population depends on the health of the environment [1, 2]. Indeed, the environmental impact of food systems has been widely studied. Dysfunctional food systems are one of the main causes of environmental degradation via greenhouse gas emissions, land conversion, deforestation, and biodiversity loss. These impacts derive from the different phases of the food supply chain, from production to consumption, including food waste [3–5]. Food and the food system are therefore of major significance when it comes to tackling climate change.

At the same time, climate change threatens public health and presents many challenges, such as reduced food and water security, increased heat-related mortality, vector- and water-borne diseases, extreme environmental events, and natural disasters [6]. Moreover, dietary patterns with high intakes of meat and meat products, fat, salt and sugar are associated with an increased risk of noncommunicable diseases (NCDs) [7]. In the diet–environment–health Syndemic trilemma, there is a profound interrelation between climate change, food production and consumption, and the health of the population (e.g., the double burden of malnutrition, overweight and obesity, and other prevalent NCDs like cancer, diabetes and cardiovascular diseases) [8–11] in different socioeconomic settings on a global scale [5]. A shift towards healthier and more sustainable diets is an imperative for the planet and its population [12].

Broad scientific consensus exists regarding the dietary patterns that the European population should adopt in order to improve its health and sustainability [13]. This means an increase in the consumption of plant-based foods (fruit, vegetables, whole grains, legumes, nuts, and seeds), and the reduction of processed and unprocessed red meat, dairy, and sugary products [5, 13, 14]. As early as 2009, the WHO highlighted the beneficial public health role of reducing the consumption of animal products, particularly in certain parts of the world [15]. Many foods that protect human health often have a lower environmental impact [16]. However, in order to make food systems healthy and sustainable, while supplying the entire population without exceeding planetary boundaries, multi-strategy solutions must be put in place. Dietary changes will be necessary, as will significant reductions in food loss and waste, and improvements in production practices [4, 5, 17, 18].

In the literature, the dietary and food system changes necessary to reduce the environmental impact of food in the European context have been widely studied. However, while several evidence-based reports exist, [3, 5, 9, 12, 19, 20] there is no consensus on global actions and policies to move us forward. Bodies like the European Commission (EC) and the United Nations (UN) as well as international pacts like the Milan Urban Food Policy Pact [21, 22] highlight the urgency of seeking strategies to transform the food system. Globally, the UN Food Systems Summit sought to deliver progress on 17 of the Sustainable Development Goals (SDGs) through a food system approach, acknowledging the food system’s connectivity with global concerns such as climate change, hunger, poverty and inequality [22]. In Europe, the EC’s ‘Green Deal’ with its ‘Farm to Fork’ strategy strives to create a green, healthy, and environmentally friendly food system [13]. Therefore, to meet the SDGs and move forward with the 2030 Agenda, new approaches are required [12, 20, 23]. While there is sufficient knowledge about these challenges to take immediate action, the implementation of a change in dietary patterns is lagging behind. For instance, for the transformation of food systems, different isolated initiatives have been launched in recent years. Among them is the creation and updating of national food-based dietary guidelines (FBDGs), which are considered a key tool for change [17]. Political and non-governmental actions, especially of a local nature and mostly in Northern Europe (e.g., updated Nordic Nutrition Recommendations), are taking place [20, 21]. These nutrition recommendations may serve as a springboard for further action towards the transformation of food systems and as a foundation for the implementation of further initiatives such as marketing regulations or the establishment of public procurement guidelines [24]. Other initiatives, such as the Milan Urban Food Policy Pact, may contribute to urban food policy, the governance of sustainable food systems in cities, and risk assessment and gap analysis for a sustainable transition [21]. In this respect, only governments possess the authority required to implement necessary changes. Furthermore, a radical transformation of food systems, which has complex social, economic, and ecological components, is required to make them sustainable, according to the Evidence Review Report of the Science Advice for Policy by European Academies, which uses an integrated systems-based approach [20]. It also mentions a lack of evidence on what works in practice, as well as the lack of national-level food policies and a fragmented EU Food Policy that lacks a unified framework and policy coherence [20].

Other sources stress the need for prioritising cross-sectoral, national, and global policy for sustainable food systems [25–27]. According to the Global Sustainable Development Report 2019 “The Future is Now”, which is focused on the science-policy-society interface, pondering how research may contribute to the 2030 Agenda, the complexity of socio ecological and socio-political concerns necessitates evidence-based dialogues on aims and remedies for “wicked” problems such as food system sustainability. National and regional levels should be included alongside the global level, whether formally or informally, especially during the implementation phase of evidence-informed policymaking [28]. For instance, the Intergovernmental Panel on Climate Change has enabled policymakers to set priorities and conduct global and regional assessments, thereby facilitating connections between multiple stakeholders, policymakers, and researchers. Consensus-based methods, such as the Delphi and nominal group techniques, may be beneficial in accomplishing this prioritisation because of their ability to generate evidence-based policy and practice recommendations from a wide range of policy process stakeholders [27, 29]. Therefore, in this three-phase qualitative study we used the nominal group technique, along with qualitative interviews and focus groups, to examine perspectives and generate consensus among nutrition, health, and environmental science experts and policymakers about the obstacles, actions, and tools required to make the WHO European Region’s diets and food systems healthier and more sustainable.

## Methods

### Study setting and context

In response to the opportunities and challenges posed by the need to transform diets, the WHO Regional Office for Europe launched the Dietary Patterns for Health and Sustainability (DPHS) project in October 2019. WHO Europe, the umbrella organisation for EU member states, convened an international panel of experts to reach a consensus on knowledge and experience that can be assimilated into lines of action.

### Overview of the conceptual framework

We adapted the Framework for Strategic Sustainable Development (FSSD) around the food system and leverage points to guide the study research questions and consensus building process [30, 31]. The FSSD comprises the following five dimensions of strategic sustainable development: 1) system (i.e., the food environment and consumer perspective of the food system); 2) success (i.e., definition of healthy and sustainable diets); 3) strategic guidelines (i.e., guidelines for prioritising actions toward success); 4) actions (i.e., concrete actions); and 5) tools (i.e., concepts, methods, and other forms of support for the decision-making and forging with the preceding levels such as monitoring and divulgence of the actions). The application of the FSSD to our study allowed us to identify and prioritise actions by experts to promote development in the direction of the stated vision of success, from the stage of conceptualisation through implementation. We investigated ways to mitigate negative repercussions and promote positive contributions through the lens of food systems [32].

**Fig. 1:**
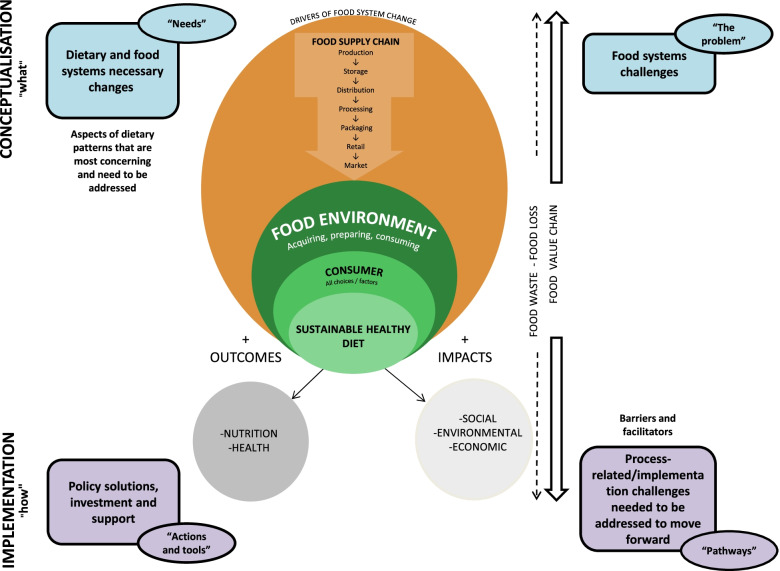
Framework for building consensus on actions for sustainable healthy diets, as well as the interconnected components and their positive effects on outcomes and impacts. Adapted from HLPE (2017) [33]

Figure 1 displays a two-dimensional framework detailing the factors to consider while developing consensus on the conceptualisation and implementation of healthy and sustainable diets. The upper part of the image depicts two components related to the conceptualisation (“what”) of these types of diets: the major issues (“the problem”) and the necessary changes (“the needs”) associated with dietary patterns and food systems. In the lower section, two additional elements pertaining to the implementation (“how”) of these diets are described: the policy solutions, investments, and support required to assist decision-makers in putting them into practice (“actions and tools”), as well as the characteristics and challenges associated with the process of implementing those actions, including the barriers and facilitators to their promotion (“pathways”).

In the centre of the figure, adapted from the High-Level Panel of Experts (HLPE) report on Nutrition and Food Systems, [33] is a schematic representation of the various interconnected components of sustainable healthy diets within the food system, such as food environments, consumer behaviour, and food supply chains, and their positive effect on outcomes and impacts. HLPE identifies the following three elements: 1) food environment, which refers to the physical, economic, political, and sociocultural context in which consumers interact with the food system to make decisions about acquiring, preparing, and consuming food; 2) consumer behaviour, which encompasses all individual and household decisions that influence personal preferences as well as the broader food environment, and 3) food supply chains, which include storage, distribution, processing, and packaging, as well as the procedures from production to sale. In addition, food waste throughout the entire food value chain is a serious and interconnected problem. To transform complex systems with these elements, activities, and actors, it is necessary to identify leverage points for a system-based approach [34, 35].

### Study design

**Fig. 2:**
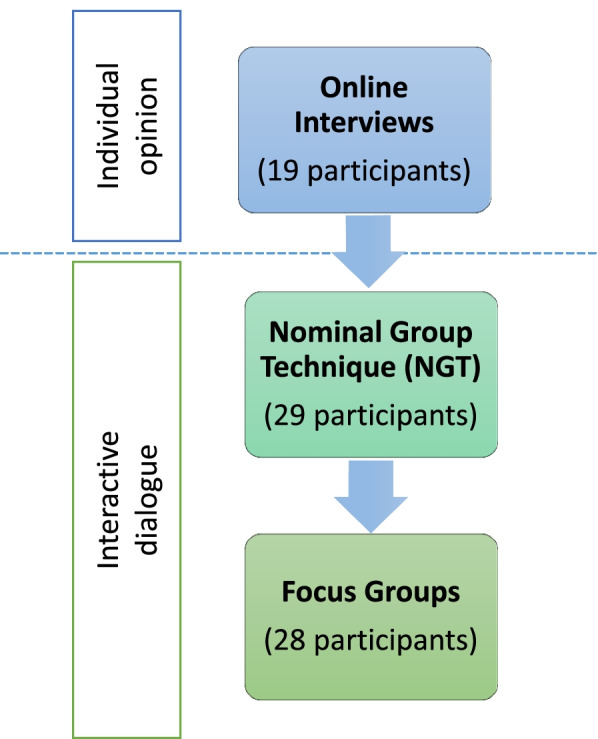
Diagram of the number of participants in each phase

A qualitative study was conducted in three sequential phases, as shown in Fig. 2. Phase 1 included online semi-structured interviews, while Phases 2 and 3, using the nominal group technique and focus groups respectively, were carried out during a two-day expert meeting workshop in October 2019 in Copenhagen organised by WHO/Europe.

### Sampling

Criterion sampling [36] was used to select participants that met at least one of the following criteria: a) researchers with experience in the field of food sustainability or public health; b) policymakers. Participants were identified in two steps. First, a list of potential participants was generated from a literature review of the topic, with feedback provided by WHO/Europe, and professionals and academics working in the field. Second, profile information for potential participants was extracted (i.e., field of expertise), and the two inclusion criteria were applied. A sample of potential participants for all three phases of this study was thus generated, although not all participants completed all three phases. Using this sample, maximum variation sampling was implemented in the semi-structured interviews to ensure the heterogeneity of participants. Nineteen participants, who had also been invited to attend the expert meeting workshop, were recruited iteratively, and interviewed. This sample size is consistent with recommendations in the literature on the number of participants needed in a qualitative study to achieve saturation [37, 38]. During the workshop, 29 participants participated in five nominal groups and 28 in four focus groups. Due to time constraints, not all workshop participants had previously taken part in the individual interviews. Nominal group technique is an effective structured brainstorming technique that enables the development of a diverse range of ideas [39]. Because the technique’s objective is to produce as many ideas as possible, some of these concepts may be mutually contradictory. However, as Boddy [39] argues, given the technique’s open and creative nature, this diversity of ideas is desirable, as even the most dissimilar ideas may “contain the grain of a good answer.” The nominal group serves to prioritise participants’ responses to questions on complex issues [40, 41] and has proven effective in generating consensus. This allows participants to share and critically discuss ideas to achieve greater clarity on the target questions [42, 43]. The nominal groups were homogeneous in terms of the participants’ field of expertise. This composition helped participants feel comfortable and speak more openly, promoting synergy and making it easier to reach a consensus. However, the focus groups were heterogeneous to ensure the greatest diversity of viewpoints. This composition made it possible to use the focus groups to confirm some of the statements that had been prioritised in the nominal groups and allowed researchers to gain further insights regarding those statements.

### Data collection

#### Phase 1: Semi-structured Interviews

The interviews were conducted in English by the principal investigator (PI) using phone calls and video calls via Google Hangouts and Skype. They lasted 30–90 min and were recorded with the prior consent of the participants. The audio recordings were transcribed verbatim. The interview guide (see Additional file 1) included 19 open questions followed up with probes and prompts to allow the interviewees to fully develop their train of thought. The preliminary analysis of the interviews provided initial insights that helped refine the questions asked in the nominal groups and the interview guide used in the focus groups.

#### Phase 2: Nominal group technique

The nominal groups were facilitated by the PI and professionals from the WHO European Office for Prevention and Control of Noncommunicable Diseases (NCD Office). The sessions lasted 120 min and were audio-recorded with the prior consent of participants. The five stages described by Harvey and Holmes [40] were implemented in each of the five nominal groups. In Stage 1, each group facilitator presented the study objectives, explained the purpose of the nominal group sessions, and ensured that all participants had signed the consent form. In Stage 2, participants were given four minutes to write down ideas on the following two open-ended questions: (1) In your opinion, what are the desirable characteristics of healthy and sustainable dietary patterns, and what points should be included in food-based dietary guidelines (FBDGs)? (2) What actions and policies need to be implemented to achieve a healthier, more sustainable food system? In Stage 3, the facilitator invited participants to share responses to the questions with the group members. Participants’ ideas were transcribed verbatim by the facilitator using a flipchart. In Stage 4, participants were asked to express agreement or disagreement with each idea. To ensure equal participation, the facilitator allocated equal speaking time to each participant. Finally, in Stage 5, participants were asked to work as a group to rank the most relevant ideas in order of importance. When all the nominal groups were finished, the rankings from the five groups were merged by the research team members in a single table. Subsequently, the ranked priority actions were organised by the research team members into 4 lines of action to be discussed in subsequent focus groups.

#### Phase 3: Focus groups

The focus groups were also facilitated by the PI and WHO professionals from the NCD Office. Four focus groups were held, each focusing on a distinct theme that corresponded to one of the previously specified four lines of action. Participants were invited to self-assign to one of the groups based on their areas of interest. Facilitators ensured that each group had a similar number of participants. Sessions lasted around 105 min on average and were recorded with the prior consent of participants. At the beginning of each session, facilitators explained the focus groups objectives, outlined the main lines of action identified during the nominal groups in response to Question 2, and read the interview guide to the participants. The guide (see Additional file 2) included 4 questions. During the session, participants were invited to express their views at will. Besides moderating the sessions, facilitators observed the participants and took field notes to help identify themes during data analysis.

### Data analysis

Data-driven thematic analysis, as described by Boyatzis [44], was used to analyse the qualitative data generated from the interviews, the nominal group technique, and the focus groups. The same four steps were used for each data source. In Step 1, all transcripts were read several times for data familiarization purposes and to identify common concepts, which were then converted into codes, and each code associated with a label, definition, and coding examples. The codes were organised in codebooks, which were discussed among the members of the research team. Any disagreements were resolved through consensus. In Step 2, two members of the research team used NVivo version 12 (QSR International, 2020) to code each transcript independently, and a consensus was again reached on any disagreements. In Step 3, a reiterative technique was used to sort, collate, and combine the codes into overarching themes, which were then checked for their relevance to the research questions. Finally, in Step 4, the NVivo ‘matrix coding query’ function was used to identify patterns in the data across participants.

### Ethics and role of the funding source

All study procedures were approved by the Institutional Review Board of the Universitat Oberta de Catalunya. The funder had no role in the study’s design, data collection, analysis, interpretation, or writing. The corresponding author had full access to all the data in the study and had final responsibility for the decision to submit for publication.

## Results

### Semi-structured interviews

The participants were interviewed online in October 2019. They included experts with one of the following five professional profiles: Environmental Footprints (*n* = 6), Food Profiling—Prioritization and Modelling (*n* = 5), General Health View (Health, Research, Policies) (*n* = 3), Communication and Policies (*n* = 3), and Government Perspective (*n* = 2). Regarding participants with a Government Perspective profile, one of these two participants was a public procurement expert, while the other was an expert in nutrition. Four main themes were identified, and a selection of participants’ quotes is presented in Table 1.

**Table 1:** Themes identified in the semi-structured interviews, with example quotes from the interviewees

Themes	Sample quotes
Theme 1. Definition of sustainable diets	“I think the definition is complete and has all the different aspects in there. I’m just wondering whether these may be too theoretical and difficult to operationalise […] we must define what we mean by environmental impacts.” (I4: Environmental Footprints) “I would say that’s a high-level definition and that’s fine, it sets the scene but now we are in a turning point in time where we actually need to be able to clarify what that actually looks like on the plate.” (I5: Food Profiling—Prioritization and Modelling)
Theme 2. Dietary and food systems necessary changes (the needs)	“It’s not possible to disseminate one universal dietary pattern on a whole region […] it is important to maintain the regional diet features in order to save cultural background or feeding behaviour.” (I16: Food Profiling—Prioritization and Modelling) “[…] we really have to change to more plant-based diets […] also reductions in food loss and waste, can do quite a bit, but again they need to be seen in combination with dietary changes.” (I1: Environmental Footprints)
Theme 3. Considerations for the promotion of sustainable diets	“Integrating food more deeply into the education system could be very powerful.” (I7: Environmental Footprints)
Theme 4. Process-related/implementation challenges needed to be addressed to move forward (pathways)	“Being inclusive and trying to engage all the relevant actors in the food system is important.” (I8: Communication and Policies) “There would be many people that don’t want to change their habits.” (I2: Environmental Footprints)

The reference after each quote indicates the number of interviewee and the professional profile

#### Theme 1. Definition of sustainable diets

Although more than half of the participants expressed their agreement with the 2010 Food and Agriculture Organization (FAO) definition of sustainable diets [45], most of them also mentioned some problems with this definition^1^ [45]. These participants highlighted the complexity of the definition, and argued that it was too theoretical and, therefore, difficult to operationalise. They suggested adding additional concepts and further clarification (i.e., defining more precisely the term ‘low environmental impact’).

#### Theme 2. Dietary and food systems necessary changes (the needs)

Most participants cited the Nordic and Mediterranean Diet as examples of healthy and sustainable diets. They also pointed out that a single pattern could not be a good fit for Europe as a whole, but existing patterns could be adapted to suit the particularities of each social context. Specifically, they considered it important to develop local perspectives that respected the culture and culinary traditions of each country. The participants also highlighted the need for dietary changes to reduce environmental impact, including a shift towards plant-based diets, reducing food loss and waste, and environmentally friendly packaging practices.

#### Theme 3. Considerations for the promotion of sustainable healthy diets

Besides regulatory measures such as policy incentives and pricing policies, participants highlighted the need to implement informative measures such as educational campaigns to promote sustainable diets.

#### Theme 4. Process-related/implementation challenges needed to be addressed to move forward (pathways)

Participants pointed out the importance of taking the entire system into account by creating a policy framework that entails a mix of solutions, while involving all stakeholders. These include the food industry, the scientific community, and government bodies.

Participants also discussed several barriers to, and facilitators in, promoting healthy and sustainable dietary patterns. Specifically, they cited population interest to fight against climate change as an example of a facilitator. Conversely, resistance to change by the different sectors involved, such as government bodies, the food industry, and consumers, was identified as the chief obstacle.

### Nominal group technique

Five groups were formed according to the participants’ professional profile: (1) Environment footprints (*n* = 7), (2) Nutrient Profiling modelling (*n* = 6), (3) Communication & Policies (*n* = 6), (4) General Health View (Health, Research, policies) (*n* = 4), and (5) Government perspective (*n* = 6). The ranking of ideas resulting from the nominal group consensus, and the sub-themes discussed for each idea and sample quotes are shown in Table 2 (for the first question) and Table 3 (for the second question).

**Table 2:** Final ranking of ideas from question 1, sub-themes included, and quotes from the participants

**Question 1.** In your opinion, what are the desirable characteristics of a healthy and sustainable dietary pattern, and what aspects should be included in food-based dietary guidelines (FBDGs)?		
Final ranking of ideas	Sub-themes^a^	Sample quotes
1.Characteristics of food-based dietary guidelines	Consideration of multiple scenarios	“Instead of single solutions, define a solution space.” (I14: NG 3 Communication and Policies)
	Be focused on evidence-based guidelines from institutional reports^b^	“The basis for these food-based dietary guidelines has to be specifically the recommendations of the EAT-Lancet Commission report because we already have the amounts of each food, each group and each food within each group.” (I18: NG 4 General Health View (Health, Research, Policies))
	Be culturally sensitive (FBDGs)	“Take preferences into account, for example, cultural preferences.” (I27: NG 2 Food Profiling—Prioritization and Modelling)
	Inclusion of standardised methodology with common tools like surveys, indicators, and outcomes	“We need to arrive at a common cause, common goals and common methods.” (I14: NG 3 Communication and Policies)
2.Core dietary aspects	Plant-based diet with a low consumption of animal products	“Include whole grains, legumes, nuts and a variety of different fruits and vegetables. Then include moderate amounts of eggs, dairy products, poultry and fish, a small amount of red meat.” (I19: NG 4 General Health View (Health, Research, Policies))
	Reduced consumption of processed food	“We should eat less processed food.” (I25: NG 5 Government Perspective)
	Moderated portion sizes	“If we target consumers, maybe it’s easier to speak in terms of servings to indicate the quantity that people should use.” (I4: NG 1 Environmental Footprints)
	Prioritised consumption of local products	“This kind of pattern should provide local food consumption.” (I16: NG 2 Food Profiling—Prioritization and Modelling)
3.Food and packaging waste	Minimal food loss and food waste	“In dietary guidelines it could be framed as a way to reuse food to avoid waste.” (I4: NG 1 Environmental Footprints)
4.Food security	Be energy-balanced and ensure nutritional intake is enough for all groups (nutritional criteria), considering the need for nutritional supplements in specific stages of life	“With these kinds of diets, you achieve the energy, adequate energy intake and micronutrients according to age, gender, according to the recommendations and to different life cycles.” (I19: NG 4 General Health View (Health, Research, Policies))
5.Socioeconomic aspects	Be affordable	“I think sustainable dietary patterns should be economically and physically affordable […] because if you can’t get some products in shops or somewhere else, it’s not possible to expect that you can include them in your dietary pattern.” (I16: NG 2 Food Profiling—Prioritization and Modelling)
	Promotion of social inclusion by developing practical guidelines (realistic/acceptability) for the different population groups to reduce inequalities	“When making the dietary guidelines, I think the socioeconomic, reducing inequalities and also global justice, is highly important. You can't recommend things that are not feasible for all population groups.” (I26: NG 5 Government Perspective)
6.Food preparation and cooking	Encouragement of cooking skills	“If you use certain types of cooking you can, in fact, lose a lot of nutrients.” (I11: NG 4 General Health View (Health, Research, Policies))
7.Biodiversity and variety	Preservation of biodiversity and assurance of the intake of a variety of food	“Even though the nutritional guidance is twice per week, in some of the Food Based Dietary Guidelines it is only once per week because of overfishing, so I think the biodiversity…” (I30: NG 1 Environmental Footprints)

The reference after each quote indicates the number of interviewee and the professional profile

*FBDGs* Food-Based Dietary Guidelines, *NG* Nominal Group

^a^ These specific components were mentioned by the participants

^b^ EAT Lancet Report, ICN2, FAO/WHO Sustainable Principles

**Table 3:** Final ranking of ideas from question 2, sub-themes included, and quotes from the participants

**Question 2**. What actions and policies should be implemented for a healthier and more sustainable food system?		
Final ranking of ideas	Sub-themes^a^	Sample quotes
1.FBDGs: legal structural global level^b^	Develop a multi-sectoral/multi-disciplinary approach	“I think it is generally important to have an inclusive approach if you want to aim for a healthy and sustainable food system […] all actors of the food system itself, whether it comes from production to manufacturing, distribution and all the way down to the consumers.” (I8: NG 3 Communication and Policies)
	Monitor	“We need to track the current consumption and impact of the current consumption and how it changed over the time.” (I6: NG 2 Food Profiling—Prioritization and Modelling)
	Regulate prices to support FBDGs	“It could be reducing the price or taxes for products that are healthy and sustainable.” (I21: NG 3 Communication and Policies)
2.Local/regional implementation^b^	Implement actions at the local/regional level	“[Speaking about implementation of policies] You have to do that at the local level but also at the national and regional level.” (I26: NG 5 Government Perspective)
3.Consumer education at all levels^b^	Carry out campaigns to increase food literacy (in schools, for instance)	“Campaigns to increase literacy in food sustainability in different settings.” (I18: NG 4 General Health View (Health, Research, Policies))
4.Advertisement to increase public awareness^b^	Run advertising campaigns	“Doing campaigns in the mass media especially TV, promoting or letting people know the real impact of our food on the environment because people are not aware.” (I2: NG 1 Environmental Footprints)
5.Healthy and sustainable public food procurement	Implement public food procurement especially in schools	“We should have that canteen as a place where we can involve the kids and try to educate them with the plate that we are serving.” (I22: NG 5 Government Perspective)
6.Food waste reduction measures	Reduce food loss and waste	“Have a focus on minimizing waste along the chain. It’s not just in the household, it’s not just in the restaurants but it’s also at the farmer’s side, it’s also at the warehouse’s side, it’s also in the delivery chain…” (I3: NG 5 Government Perspective)
7.Food production measures (technology) and food reformulation	Develop sustainable production systems	“This way you would actually have people who are not interested in sustainability, randomly picking products that are more sustainable. That is why it is so important that we support the food producers in making more sustainable products.” (I17: NG 3 Communication and Policies) “The manufacturers in themselves have to aim continuously for a good nutritional profile for the foods that are marketed.” (I8: NG 3 Communication and Policies)
8.Food labelling	Rank foods by a FoP interpretative labelling system for footprints	“Also measures to increase literacy could be to implement a colour system to rank foods regarding the environmental impact.” (I18: NG 4 General Health View (Health, Research, Policies))

The reference after each quote indicates the number of interviewee and the professional profile

*FBDGs* Food-Based Dietary Guidelines, *NG* Nominal Group, *FoP* Front of pack

^a^ These specific components were mentioned by the participants

^b^ In items 1–4, a requirement to define global/local responsibilities and build networks was stressed

Question 1: What are the desirable characteristics of healthy and sustainable dietary patterns; and what points should be included in food-based dietary guidelines (FBDGs)?

Participants ranked seven characteristics. The first was general recommendations, including the importance of creating guidelines using evidence-based principles, and several published reports [3, 9, 19] that could be used to generate those principles were cited. They also discussed the importance of strengthening the existing research since most actions in the implementation process are not grounded on a solid knowledge base, and they cited the need to study multiple environmental indicators, such as water use, land use, nitrogen, and greenhouse gas emissions. Reference was also made to the need to monitor current dietary patterns. Second, participants pointed out the need for dietary changes, including prioritizing plant-based diets with low consumption of animal products, reducing consumption of processed food, moderating food portion sizes, and promoting the consumption of local products. Third, food waste reduction was mentioned as another critical aspect and the need to include a recommendation to prevent it in the FBDGs was stressed. The need for diets that ensure food security and meet individual energy and nutritional requirements was ranked fourth. Participants mentioned that specific guidelines should be created for different population groups, including children, women, and the elderly. Fifth, they highlighted socioeconomic aspects such as social and cultural acceptance of diets, accessibility of food, and equity of distribution, as essential to healthy and sustainable diets, and they stressed the need to consider the cost of food to avoid inequalities. Sixth, participants noted the need to consider good cooking practices to avoid losing nutrients, and seventh, they highlighted the preservation of biodiversity as necessary for a healthy and sustainable diet.

Some debate took place during the generation of statements for the first question, since approximately one third of the participants considered that processed foods were acceptable if food safety, nutritional quality and low environmental impact was assured. On the recommendation for local foods, several participants from nominal group 1 (Environmental Footprints) questioned the inclusion of local foods as an element of healthy and environmentally sustainable diets globally due to the lack of strong scientific evidence supporting the consumption of local food as having less environmental impact and better nutritional quality compared to imported products.

Question 2: What actions and policies need to be implemented to achieve a healthier, more sustainable food system?

The ranking of priorities included eight actions, including some considerations for the implementation of these actions. Global actions related to FBDGs concerning legal structure were ranked first. Most participants emphasised the importance of simultaneously implementing a mix of policies, which should be aligned and coherent, and involve all sectors of the different disciplines involved. To achieve this, they highlighted the need to seek multiple solutions, since no single solution is sufficient. Participants mentioned the existence of trade-offs and the importance of quantifying them (e.g., impact indicators). Second, local, and regional implementation of the actions was also considered as a priority. Third, many of the participants referred to the need for educational measures targeting consumers and implemented at all levels, including food and nutritional education, the development of the FBDGs, carrying out mass media campaigns, and considering front-of-pack labelling (FoPL). Fourth, was the use of advertising to increase public awareness. Participants highlighted the need to regulate industry media campaigns to restrict the promotion of unhealthy and unsustainable food and promote healthy eating habits. Fifth, the implementation of public food procurement in schools and in other settings was stressed by some participants. A couple discussed the usefulness of public food procurement in schools as an educational measure for children. Sixth, like in question 1, reducing food waste throughout the whole food chain was also considered a priority. Seventh, several participants noted the importance of implementing production measures that help producers deliver healthier and more sustainable food, increasing its accessibility and availability. Some stressed the importance of creating and supporting new technological tools to deal with food waste. Others mentioned the need to reformulate food products to improve their nutritional quality. Finally, participants noted that labelling could help to inform consumers and help them make better choices. It was also mentioned that environmental impact factors should be included in labelling, in addition to information on nutrition composition and health claims.

### Focus groups

The following focus groups were formed each focusing on one of the four action lines arising from the nominal group prioritization: Focus Group 1 Food Supply (e.g., setting food prices to support FBDGs) (*n* = 8), Focus Group 2 Food Demand (e.g., launching campaigns to increase consumers’ food literacy) (*n* = 9), Focus Group 3 Public Procurement (*n* = 6), Focus Group 4 Food and Packaging Waste/Food Production (Technology, Water Crisis) and Food Reformulation (*n* = 5). As explained in the methods section, each participant selected the group in which they wished to participate (see Additional file 3). Themes discussed by the focus groups along with sample quotes are shown in Table 4.

**Table 4:** Themes and subthemes identified in the focus groups

Themes	Subthemes	Sample quotes
Theme 1. Actions to overcome the challenge of implementing a more sustainable and healthier food system	Food composition	“So, giving principles and guidelines from the ready guidelines that we have from FAO, and you may be doing for the industry some specific guidelines that could be useful for reformulating, taking into account all the aspects we already agreed in the recommendations of the reports.” (I38: Communication and Policies)
	Food retail	“We should find ways to influence also the parents more directly, […] to influence consumer in general, […] for instance, changing completely the way food is displayed in supermarket to incentivise a different type of consumption.” (I4: Environmental Footprints)
	Food provision	“Food-based dietary guidelines which are both healthy and sustainable in each region.” (I10: Food Profiling—Prioritization and Modelling)
Theme 2. Characteristics of the action process for implementing a more sustainable and healthier food system	Multi-stakeholder	“Cross-sectoral working group of specialists that know a lot in their area, because then we would learn from each other.” (I3: Government Perspective) “I see like at least three […] but can be like other stakeholders in three areas. One is education, second one is health, and then is like the social security […] perhaps the challenge we face is that the three don’t cooperate, don’t collaborate, don’t speak to each other…” (I30: Environmental Footprints)
	Evidence-based	“You would create some evidence, you would publish some papers, and then you would start working towards some kind of a goal.” (I29: Communication and Policies)
	Adapted to the context	“The difficulty is we cannot use the same standard everywhere and there is a need for adaptation between cities, regions, countries depending on the food system.” (I21: Communication and Policies) “I think it’s each country has so much as a different reality that you cannot have a model that fit for all.” (I20: General Health View (Health, Research, Policies))
Theme 3. Resources needed to implement specific actions	Resources	“Knowledge, skills and also materials and infrastructure, they are all needed.” (I26: Government Perspective)
	Obstacles to implementing actions	“I’ve seen some limitations in some countries regarding the budget funding.” (I21: Communication and Policies)

The reference after each quote indicates the number of interviewee and the professional profile

#### Theme 1. Actions to overcome the challenge of implementing a more sustainable and healthier food system

Participants highlighted the following actions as the most relevant: generating guidelines such as food production and food composition or reformulation guidelines (standards to improve the nutritional quality of food), implementing food environment policies (e.g., provision and disposition of foods in public and private settings, price, and availability of foods), and developing and updating the FBDGs.

#### Theme 2. Characteristics of the action process for implementing a more sustainable and healthier food system

Participants mentioned the alignment and coherence of policies as a key part of the implementation process, involving stakeholders from different sectors. These participants also mentioned the lack of cooperation between stakeholders as a possible obstacle, citing as important needs, improved communication between stakeholders and agreement on common goals. Policymakers and public authorities (political support at national and local level), scientists, international organizations, food suppliers, civil society, and educators were the stakeholders most often cited by participants as those who needed to be involved in the action process. Besides, capacity building (e.g., training of all the actors around the food chain, teachers, among others) and implementing evaluation and monitoring systems (e.g. monitoring SDGs, evaluate guidelines, monitoring public procurement and food waste, and mapping monitoring initiatives) were highlighted. Participants also mentioned the need to define and carry out the implementation process using evidence-based knowledge. Other participants stressed that the action implementation process should be adapted to the national, regional, or local context and to the cultural background. Some pointed out the difficulties involved in adaptation, since a single standard model is not suitable for all settings.

#### Theme 3. Resources needed to implement specific actions

The most frequently mentioned resources that could facilitate implementing actions were investment (for instance, investment to implement educational measures at different levels), the existence of evidence and open-source data, the creation of networks of experts, develop a regulation system (e.g., regulate marketing), work on consumer acceptance and behaviour, the use of standardised methodology, and the creation of health and sustainability logos. Some of the participants who mentioned investment as a resource also referred to a lack of funding as an obstacle to implementing the actions.

## Discussion

**Fig. 3:**
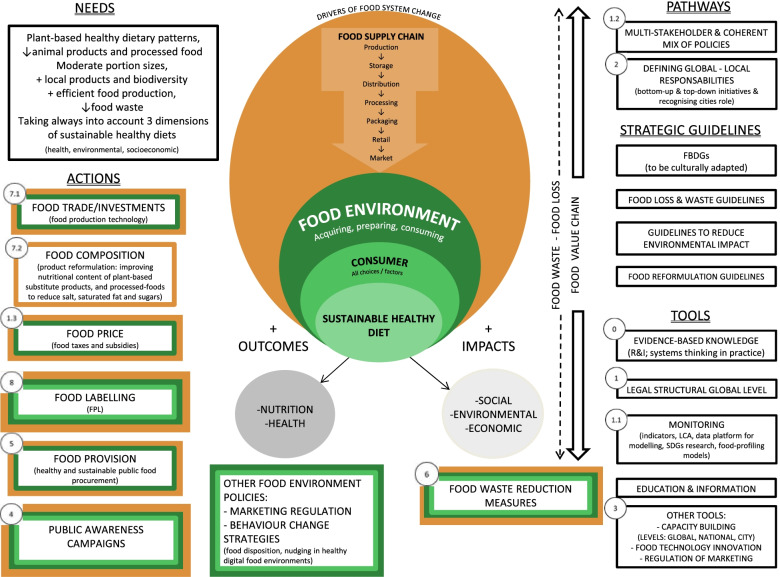
Needs, actions in the different policy domains, tools, strategic guidelines and pathways for sustainable healthy diets. The numbers in the figure represent the prioritisation in the nominal groups, ranging from a global legal structural level to less prioritised specific measures, such as food labelling, to a cross-cutting feature of evidence-based knowledge. The colour of each action corresponds to its position in the food value chain

Sustainable healthy diets have low environmental impact, contribute favourably to nutrition and health outcomes, as well as social, environmental, and economic impacts, and hence to the health of the planet as a whole. While food and nutrition policies should be at the heart of healthy and sustainable diet promotion, sustainability has not yet been fully integrated into policies [46]. Previous literature has set the groundwork for building up a healthy and sustainable diet conceptualisation and for determining the path to success [46]. However, traditional approaches have sometimes been limited because food systems are complex and require a more comprehensive and coordinated perspective [32], as well as efficient global–local progress due to the urgency of environmental concerns [26]. There are few practical examples of the implementation of healthy sustainable diet promotion in European countries. There is a call for evidence-based dialogues on solutions for problems such as food system sustainability [28]. Parallel to this, expert consensus from a variety of stakeholders, including researchers, public health experts, and policymakers, has guided policy in a number of areas [29], recognising the value of policymaker expertise, from global and local levels, particularly for policy implementation [28]. In order to garner political and popular support for future actions, the present study sought to address the lack of a coherent plan for healthy and sustainable diets in Europe. The conceptualisation and implementation of the progress toward healthy and sustainable diets was determined from a consensus-based method with key international experts and policymakers in nutrition, health, and environmental sciences, as well as a food system approach analysis. This strategy provided a shared understanding of sustainable healthy diet components and potential solutions. Figure 3 outlines the needs, actions in the various policy domains, instruments, strategic guidelines, and routes for sustainable healthy diets, based on the study’s findings. In both the individual interviews and the workshop, participants emphasised the necessity for a multi-stakeholder approach and simultaneous implementation of aligned and coherent policies at the local and national levels. The findings demonstrate the need for a shift in eating patterns toward sustainability; plant-based diets low in processed foods, moderate portion sizes, local products, and an emphasis on protecting biodiversity. In addition, participants called for more efficient food production, considering the health, environmental, and socioeconomic elements of food sustainability, and a reduction in food waste along the food value chain. Employing evidence-based knowledge, establishing a legal structural worldwide level, utilizing rigorous monitoring methods, and providing education and information were among the tools identified. Other factors mentioned included capacity building at the global, national, and local levels, innovation in food technology, and marketing regulation. Strategic guidelines are essential, with the most important being the incorporation of sustainability into FBDGs and the emphasis on cultural adaptation, as well as other pertinent recommendations regarding food loss and waste, to reduce environmental effect and food reformulation. Food price or fiscal measures (e.g., subsidies and taxes), public awareness campaigns, food provision (e.g., healthy and sustainable public food procurement), food waste reduction measures, food trade or investments (e.g., food production technologies), and food composition and labelling of sustainable and healthy diet options were ranked in order of priority in the policy domains shown in Fig. 3.

On the first dimension of conceptualisation, “the problem”, most of the participants in our study agreed on the FAO definition of food sustainability [3, 45], while some mentioned it was complex and suggested adding clarification for certain concepts. This is consistent with a number of authors that have noted the lack of a universal definition of food sustainability [20]. Although the concept is widely used by a variety of institutions and communities, it is often based on a narrow definition that focuses exclusively on its environmental component rather than a more comprehensive definition that acknowledges its multidimensionality, including the following four key features of sustainability: nutritional, social, economic, and ecological [20, 47]. For instance, the Scientific Opinion Board towards a sustainable food system of the EU, acknowledged a broader definition and argued that all food policies should ensure the social, economic, and ecological features of sustainability [48]. Besides, food system components are not isolated. Food system reform to support healthy and sustainable diets can only happen by considering its interrelated components and food choice drivers, and find intervention spots within it [33, 49].

Our findings on the consensus on “needs” show that efforts should be made to switch eating patterns towards sustainability considering health, environmental, and socioeconomic aspects, in addition to improving food production efficiency, reducing the environmental impact of food production, and reducing food waste along the food value chain is also important. The core dietary patterns that require attention are plant-based and low in animal products and processed food, aim for moderate portion sizes, promote local products, and foster biodiversity. This agrees with the current evidence on the topic [46]. Current European dietary patterns (especially for the younger, urban and/or low-income generation) have undergone ‘Westernization’. This has been due to the socioeconomic and cultural factors during food transitions, where consumption of animal proteins has increased markedly, as have empty calories from refined added sugars, fats, alcohol, and sugary drinks [50, 51]. To lessen the environmental impact of dietary patterns, the need for a shift to plant-based diets, without the necessity of eliminating meat entirely, was emphasised, in agreement with scientific evidence [18, 52]. Significant reductions in greenhouse gas emissions can still be achieved [53]. According to the Intergovernmental Panel on Climate Change, animal foods produced in sustainable systems with low greenhouse gas emissions could be part of the solution [5]. Excessive red meat intake has a negative impact on public health, [9, 54] and greenhouse gas emissions and other environmental footprints are generally higher in the production of meat than in that of any plant food [55]. Given the cultural dimensions of food consumption, public health recommendations and environmental considerations [56] have to be adapted to the traditions, culture, and gastronomy of every region in Europe, and this was also a major concern for the experts. In Europe, some dietary patterns currently being analysed may result in solutions that are more sustainable within each particular context, namely the Nordic, Mediterranean, and Lowlands diets [57–59]. Several systematic reviews have been published evaluating diets alongside the environmental and health impact [50, 60]. Scientific evidence indicates that healthier dietary patterns are based on plant-based foods and lower in foods of animal origin [14, 61]. Sustainable food sources and crops are also stressed [46]. However, besides shifting dietary patterns towards more plant-based diets, a reduction in ultra-processed food was stressed, given the associations with adverse health outcomes [62]. Ultra-processed foods are generally rich in added sugars, salt and/or fat, often containing little or no whole foods, as the NOVA classification describes [63]. Whereas plant-based diets, specifically if high-quality plant foods are present, have been systematically associated with lower risk of NCDs [64]. Some plant-based ultra-processed foods, such as meat analogues, may have a low environmental impact, but more research on the health effects derived from their consumption is needed [65]. For environmental, health and animal welfare concerns, plant-based diets are a trending topic facing challenges [66], such as food acceptability and the risk of nutritional deficiencies in some who follow more restrictive diets [67]. Additional research is needed on alternative protein sources suitable for preventing micronutrient deficiencies and with low environmental impact [68].

The consensus on the guidance to deliver healthy sustainable diets and the needs revealed the usefulness of national FBDGs as a tool for promoting healthy and sustainable diets that are tailored to each country’s unique characteristics. It was considered necessary to update existing guidelines, or to create and implement new ones where none exists. According to Martini et al. [69] approximately 20% of more recent the European FBDGs (published from 2011) incorporate environmental impact, while longer-standing FBDGs (published before 2010) do not consider the environmental impact. In our study, expert consensus included the recommendation that FBDGs should do more to ensure that dietary patterns reflect sustainability objectives, a recommendation that is backed up with substantial evidence [61, 70]. For example, participants recommended that FBDGs accommodate or advocate a more plant-based diet, which might save a large quantity of greenhouse gases, according to the literature [71]. A key finding from the nominal groups was the perceived need for general FBDGs recommendations that (a) considered multiple scenarios, (b) were evidence-based as stated elsewhere [3, 9, 19], (c) were culturally adapted, and (d) used a standardised methodology with common tools. Besides the plant-based food base, other aspects considered relevant for the guidelines were food safety, sufficient nutritional intake for all age groups, affordability, and the need to consider food supplements at specific life stages and include cooking tips and ways to reduce food and package waste. These results are consistent with an existing conceptual framework for the future development of FBDGs in Europe [70] which claims that in numerous countries, environmental considerations were made in addition to food safety, dietary habits, and preparation. Likewise, our findings are consonant with a Review of FBDGs of National Guidance for Children, Adolescents, and Women which concluded that the food supplementation was only considered necessary in special situations and stages of life [28, 72], and with the claim, reported elsewhere, that the FBDGs must pay attention to sociocultural issues, such as rapidly changing food habits [73]. Other guidelines around food loss and waste, environmental impact reduction, and food reformulation were deemed necessary, and the literature goes some way to address them [46].

In regard to the implementation dimension of our framework, specific actions mentioned were public awareness campaigns, food provision (e.g., healthy and sustainable public food procurement), food waste reduction measures along the food value chain, food trade or investments (e.g., food production technologies), reformulation and labelling sustainable and healthy options (e.g., FPL and menus). Several studies have examined these policy instruments for healthy and sustainable food consumption, ranging from information provision or communication, economic or fiscal activities, and regulatory measures to behavioural modification techniques [74]. Existing policies, primarily target producers and consumers, whereas evidence suggests that efforts should be made to target food processing and retail stages [48].

Participants highly prioritised actions focusing on food supply according to the legal structural global level of the FBDGs (pricing regulation such as subsidies, incentives, taxes, etc.). A narrative review of the regulatory governance factors that influence food and nutrition policies aimed at preventing diet-related noncommunicable diseases reveals a range of regulatory designs used in both policy formulation and implementation, including state command-and-control regulation of taxes and menu labels, quasi-regulation of sodium reformulation, and co-regulation and industry self-regulation of food marketing policies [75]. The criticism of quasi-regulation and industry self-regulation is for its optional nature, industry’s lack of independence, and deficient monitoring and enforcement. The best practices on nutritional policy seem to point towards comprehensive policy objectives with accurate standards monitored and enforced [75]. For achieving change towards food sustainability, another Scientific Opinion Board also claimed, that core drivers are necessary fiscal and regulation policy measures [48]. However, fiscal instruments are controversial, particularly taxes, as industry lobbies claim that they could exacerbate inequality [76]. Evidence suggests this is not the case, and taxes could be used to tax unsustainable unhealthy foods and subsidise sustainable healthy foods [77, 78]. In addition to the establishment of a global legal structure, the tools that emerged from our study range from the use of evidence-based knowledge and robust monitoring tools (e.g., sustainability indicators, life cycle assessments, data platforms for modelling, SDGs research, food-profiling models) to education and information. Even with certain advancements in monitoring metrics and indicators, there is still a lack of universal indicators to determine efficacy or progress and success [79]. Other instruments mentioned included capacity building at the global, national, and local levels, innovation in food technology, and marketing regulation. All of these instruments have been identified elsewhere and aid to accelerate change towards the vision [46]. Consequently, it is essential that national governments assist domestic food producers in reducing their environmental footprint. [28]

The need to explore public food procurement potential was pointed out by participants. Some authors consider it relevant to inform practices based on sustainability criteria from public procurement schemes within food service [80]. For example, initiatives such as the Baltic Sea Region which developed a Sustainable Public Meal Toolkit provides experience-based counselling on strategies and activities for sustainable public procurement and catering services [81]. The European Parliament described sustainable public procurement as “a process by which public authorities seek to achieve the appropriate balance between the three pillars of sustainable development—economic, social and environmental—when procuring goods, services or works at all stages of the project” [82]. A step further is green public procurement, which aims to decrease environmental impact rather than just apply compensatory measures [82]. Research into sustainable public procurement suggests that public food catering services influence the food sector [46, 83] and that there is room for improvement for aligning with the procurement directive guidance towards sustainability for public procurement [82].

In terms of influencing demand, experts highlighted the need of educating consumers and using advertisements to increase public awareness which were reported to be complementary measures. It was agreed by experts, and stated in the literature, [20] that providing information is unlikely to elicit change if not accompanied by ‘tougher’ measures such as legislation and taxation [84]. This justifies why legislation will be critical over education. In addition, according to the Scientific Opinion Board towards a sustainable food system [48], it is required to combine regulatory, financial, behavioural, information (certification and labelling), communication (awareness campaigns) and education measures. However, it is claimed that those measures may be insufficient on their own, as consumer choice is influenced by additional factors such as preferences, advertising, and pricing [48]. While voluntary consumer activities may be beneficial, they should not be depended upon as key drivers of change [48, 85–87]. Information-designed measures have a role as instruments for promoting healthier and sustainable food choices and raising consumer awareness in Europe [88]. For instance, early-age education on food, health and sustainability is needed to change eating patterns of new generations. It is also essential to monitor the marketing of ultra- processed foods [28]. FoPL which incorporates food ecological footprints was suggested by many experts as a tool to increase food literacy in relation to environmental impact. The metrics to guide such policy actions are controversial and contested in the literature [85–87]. Some participants stated that rating ‘food healthiness’ should be based on evidence of food-based research including the level of processing as reported by epidemiological and mechanistic studies [63]. Others declared that it can be rated using nutrient profiling, despite the core principle underpinnings FBDGs that a food’s health potential is determined by more than the sum of the nutrients it contains [63]. Standardised models to assess the health and/or environmental credentials of foods are being developed, for example the EcoScore in France. Current models focus mostly on water and carbon footprint when assessing the environmental impact of a food product/diet. This ignores the assessment of other food-derived environmental effects such as eutrophication, land use change or biodiversity loss, among others. It is not desirable that consumer education actions and FoPL could place undue responsibility (and potentially blame and shame) on consumers compared to other actors within the food system and beyond [87]. Moreover, evidence seems to show that consumers who already care about environmental issues are interested in sustainability labels [89]. Additionally, sustainable credentials often give foods a ‘health halo’, as with plant- based foods [90]. Better common understanding of sustainable healthy nutrition is needed [46]. Other food environment measures related to behaviour change are food disposition and nudging, including in the digital food environment, [91] where more research to determine how we can best utilise these systems to support public health nutrition is needed [92].

Other actions at different points of the food chain that were reported as being important were food and packaging waste and investing in food production technologies and reformulation towards more sustainable and healthier food production. The reduction in food waste was also highlighted by the participants, both individually and in groups, as the need for strategic food loss and waste guidelines, even though policy specific solutions related to this topic did not emerge. The Lancet Commission recommends to reduce food loss and food waste by 50% by 2030 [12]. Agreeing on a definition of food loss and waste is complex, with different agencies including different food system stages. FAO and UNEP report food losses as what is lost in production, not including retail, whereas food waste includes retail and consumption level [3, 93]. The literature suggests pricing incentives and information provision, which may motivate the private sector to decrease food loss and waste for their own benefit [3]. Governments and businesses may take measures to prevent post-harvest losses, especially in critical loss points, and urban policies should encourage consumer-level food waste reduction initiatives [94]. The Science Advice for Policy by European Academies report recommendation stated to act beyond households and develop a more circular food system [20, 93] In agreement with the New Climate Economy Report, while significant resources will be required to develop food waste prevention programs throughout the supply chain, the economic and environmental benefits of food waste reduction are significant, and the costs of inaction are severe [94]. However, food waste is not regarded as a principal path to sustainable healthier nutrition in isolation [95].

With reference to the pathways, barriers and facilitators, a range of process-related aspects or implementation challenges that need to be addressed to move forward were raised by the experts. Regarding the food system transformation process, participants emphasised the requirement for taking a multi-stakeholder approach, involving all actors, from production to consumption. Emphasis was also placed on an aligned and coherent implementation of different policies. Thus, responsibility for it would fall to the various stakeholders and not only to consumers but a wide range, from food producers and food industry, the scientific community to government bodies [96]. This is in line with the recommendation from the European Commission’s “Towards a sustainable food system” report that reiterate the need to ensure a fully integrated approach to bring about a sustainable food system. [48] There are many key food actors in the food policy landscape in Europe who could affect consumer behaviour regarding sustainable healthy diets, from an individual level to community, governmental and global level, including public health organizations and consumer organisations. [46] Other sources have added a requirement for coordination besides the interdisciplinary and inter-sectoral perspectives. [20] Moreover, other food policy literature suggests good governance and political support are critical for multisectoral implementation of policies for health and wellbeing [97], and so far has been inadequate [74]. For policymakers, the sustainability concept is complicated and requires those intersectoral reactions and thinking, as mentioned earlier. Some examples of experiences in the Region exist in Sweden, [98] Germany, [99] and Netherlands [100] which committed to implement healthy and sustainable food consumption and production. However political processes that examine problems and potentialities in health and sustainability within strategic food policy action plans, have not been properly established [101]. At an international level, there is the European Union’s regulatory framework providing information to consumers, and the Common Agricultural Policy (CAP), the foundations of the food and feed legislation, both at Union and at Member State levels [46]. More traditional food security programs had a tendency towards a production-focused approach. Currently, incorporation of environmental concerns within the CAP is essential. Supranational level coordinated actions are required, as food items are often produced by multinational companies [46]. Defining global and local responsibilities was stressed by the participants, with a combination of bottom-up and top-down initiatives as described elsewhere [102, 103]. This finding is in line with the Scientific Opinion Board towards a sustainable food system [48] which recommended that a capacity for transformation be established through the design of both relevant EU policies and national, regional and local initiatives. Apart from network development, it was emphasised by experts that cities are critical for action implementation. For instance, Urban Food Policy Pact Global Forum, [21] demonstrates how cities can play a critical role in governing food systems in order to achieve a sustainable transition. Together with the lack of political support, one of the major perceived barriers is the need for changes in behaviour across food environments, not only by consumers but also producers and distributors [28]. It may imply the necessity of challenging socio-cultural norms and practices and, at the same time, facilitating food availability and accessibility by ease and affordability for actors for responsible decisions concerning sustainable and healthy diets [28].

For future work on implementing evidence-based policies and interventions, the identified actions could be validated with consumers and all stakeholders and its cost-effectiveness analysed [104]. This is especially critical for FoPL and consumer education. The effectiveness of actions to improve healthy diets has been reviewed elsewhere and shows strong evidence on pricing strategies and school public food procurement policies, less evidence on the effectiveness of mass media campaigns, and inconclusive evidence for changing food environment (food availability or accessibility) and for food labelling [105]. The latter, therefore, should be provided in combination with other interventions such as mass media campaigns and education [105]. Additional research is needed to evaluate the impact of actions to meet the SDGs and Eat-Lancet Commission’s policy range from doing nothing to eliminating the choice.

Additionally, the interaction between structural and commercial determinants of health with individual factors is relevant for developing public health nutrition strategies that should be considered [106]. This is particularly pertinent if complex interconnections between climate change, inequity and nutrition outcomes are acknowledged [107]. Recognizing the imbalances in the excessive consumption by some to under-consumption by others is important, as equity is a fundamental component of climate change, nutrition and global health research [107]. While prevention and management of conflicts of interest in food policies and programs within countries is necessary, [2] making use of a social marketing approach could enhance mass media campaigns for the adoption of healthy and sustainable diets [106].

As for the implications and future research, results obtained through consensus on actions for developing sustainable healthy diets from key experts in the field will serve to inform policy, along with existing scientific evidence. Research was deemed an essential transversal element in the policy implementation process as was highlighted by the participants. In this context, some existing evidence-based reports [3, 9, 19] were mentioned as a starting point for developing recommendations. As the EC’s “Towards a Sustainable Food System” expert report expresses; initiatives at all levels should be evidence-based and integrate expert advice [48]. In fact, the goal of the present project was to prioritise actions based on expert opinion and consensus, which formed the basis for the recent report on the key workstreams in the WHO European Region on Sustainable Healthy Diets [108]. This is needed to support countries in their requests for more clarity on how to change dietary patterns and facilitate knowledge-sharing. As well as, to allow researchers collaboration to share databases and harmonising protocols and procedures for monitoring.

These results are worth future discussions involving different stakeholders to further explore the issues highlighted by the experts. A broader range of disciplines and countries in the WHO European Region should be included. Given the discrepancies among participants in relation to local foods and processed products, such topics are particularly important. Indeed, it would allow better identification of the problem and solutions defined in a comprehensive, evidence-based, approach that policymakers can follow. Several case studies from across the European Region would further provide examples of good practices that could be replicated, with a subsequent assessment of their impact. In consonance with scientific expert groups in the EU emphasise the need of a learning-focused policy approach and governance structures through pilot initiatives, assessing their suitability for broader adoption [48].

The study has several strengths and limitations. One of the strengths of this study was the participatory dialogue based on the workshop that brought together relevant experts in the field with diverse profiles from different countries across Europe. The development of expert consensus is another defining strength of the study. In the food sustainability field with an enormous and disparate evidence base, expert consensus is highly appreciated [97]. The nominal groups revealed the existence of some controversy in relation to what constitutes a healthy and sustainable diet. Specifically, there were some discrepancies in terms of local and processed foods. Despite the controversies, recommendations which were broad but adaptable to different cultural contexts were formulated through a consensus from various points of view. Facilitating public health researchers and policymakers to employ systems thinking approaches will aid multi stakeholder cooperation and policy coordination to effectively tackle future challenges [32]. The main limitations of this study were the small sample size and the underrepresentation of some professional profiles of the experts such as the “Government Perspective” profile, as well as a focus on only a limited number of European countries. Interviews were conducted with 19 of the 32 participants only. Due to scheduling conflicts, only two participants who identified themselves as having a ‘Government Perspective’ were able to be interviewed. Seven participants took part in only one of the two stakeholder dialogue phases due to scheduling issues. If more time had been available to hold group sessions, deeper reflections on the specific topics could have been reported.

## Conclusion

In an effort to fully integrate sustainability into healthy food policies, [46] a consensus-based method with key international experts and policymakers in the fields of nutrition, health, and environmental sciences was used. This, in combination with food system approach analysis, established the conceptualisation and implementation for the progress toward healthy and sustainable diets in Europe. A collective understanding of the interconnected components of sustainable healthy diets contributed to prioritising potential solutions: actions in the different policy domains, tools, strategic guidelines and pathways for sustainable healthy diets. Results agreed with existing evidence on political processes, actions, and experiences in the field of sustainable and healthy eating [79]. Future inclusive dialogues between scientists, policymakers and other stakeholders including the different actors in the food system and beyond are needed to provide solutions to face the challenge of a resilient and sustainable food system for planetary health. Implications for future policies require research and should account for political, social, and economic dimensions, as well as trade-offs, to holistically change complex food systems [3, 5, 9, 17, 19, 20, 109]. Promoting a common food policy framework at the various levels of governance was deemed essential, with city governments vital as food policy actors. Finally, pooled analysis of experts’ opinions alongside scientific literature gave an indication of potential research and key actions needed in Europe [108]. This work will support action in Member States, not only considering human health but also environmental health, to urgently join efforts to promote a healthy and sustainable food model across the WHO European Region.

## Supplementary Information

**Additional file 1: Table S1.** Script of questions asked during the semi-structured interview.

**Additional file 2: Table S2.** Questions asked in the focus group session.

**Additional file 3: Table S3.** Number of participants in each focus group, by professional profile.

**Additional file 4: **List of members of the DPHS project Expert Group.

## Data Availability

The datasets generated and/or analysed during the current study are not publicly available due to the fact that participants did not give consent for their data to be shared in this manner, but requests for anonymised data can be made to the Principal Investigator of the study, Anna Bach-Faig (abachf@uoc.edu).

## Footnotes

1 “Sustainable diets are those diets with low environmental impacts which contribute to food and nutrition security and to healthy life for present and future generations. Sustainable diets are protective and respectful of biodiversity and ecosystems, culturally acceptable, accessible, economically fair and affordable; nutritionally adequate, safe and healthy; while optimizing natural and human resources”.

## References

[1] Meybeck A, Gitz V (2017). Conference on ‘sustainable food consumption’ sustainable diets within sustainable food systems. Proc Nutr Soc.

[2] Whitmee S, Haines A, Beyrer C, Boltz F, Capon AG, De Souza Dias BF, Ezeh A, Frumkin H, Gong P, Head P (2015). Safeguarding human health in the Anthropocene epoch: report of the Rockefeller Foundation-Lancet Commission on planetary health. Lancet.

[3] Food and Agriculture Organization of the United Nations, World Health Organization (2019). Sustainable healthy diets. Guiding principles.

[4] Galli A, Iha K, Halle M, El Bilali H, Grunewald N, Eaton D, Capone R, Debs P, Bottalico F (2017). Mediterranean countries’ food consumption and sourcing patterns: an ecological footprint viewpoint. Sci Total Environ.

[5] Intergovernmental Panel on Climate Change: Climate Change and Land: An IPCC Special Report on climate change, desertification, land degradation, sustainable land management, food security, and greenhouse gas fluxes in terrestrial ecosystems. 2019.

[6] Kim B, Neff R, Santo R, Vigorito J (2015). The importance of reducing animal product consumption and wasted food in mitigating catastrophic climate change.

[7] Cena H, Calder PC (2020). Defining a healthy diet: evidence for the role of contemporary dietary patterns in health and disease. Nutrients.

[8] Chopra M, Galbraith S, Darnton-Hill I (2002). A global response to a global problem: the epidemic of overnutrition. Bull World Health Organ.

[9] EAT Lancet Commission (2019). Food planet health. Healthy diets from sustainable food systems.

[10] Nishida C, Uauy R, Kumanyika S, Shetty P (2004). The Joint WHO/FAO expert consultation on diet, nutrition and the prevention of chronic diseases: process, product and policy implications. Public Health Nutr.

[11] Swinburn BA, Kraak VI, Allender S, Atkins VJ, Baker PI, Bogard JR, Brinsden H, Calvillo A, De Schutter O, Devarajan R (2019). The global syndemic of obesity, undernutrition, and climate change: the lancet commission report. Lancet.

[12] Bodirsky BL, Dietrich JP, Martinelli E, Stenstad A, Pradhan P, Gabrysch S, Mishra A, Weindl I, Le Mouël C, Rolinski S (2020). The ongoing nutrition transition thwarts long-term targets for food security, public health and environmental protection. Sci Rep.

[13] European Comission (2021). Farm to fork strategy: for a fair, healthy and environmentally-friendly food system.

[14] Willett W, Rockström J, Loken B, Springmann M, Lang T, Vermeulen S, Garnett T, Tilman D, DeClerck F, Wood A (2019). Food in the Anthropocene: the EAT–Lancet Commission on healthy diets from sustainable food systems. Lancet.

[15] Chan M (2009). Cutting carbon, improving health. Lancet.

[16] Clark MA, Springmann M, Hill J, Tilman D (2019). Multiple health and environmental impacts of foods. Proc Natl Acad Sci USA.

[17] Loken B, Declerck F (2020). Diets for a better future: rebooting and reimagining healthy and sustainable food systems in the G20.

[18] Springmann M, Spajic L, Clark MA, Poore J, Herforth A, Webb P, Rayner M, Scarborough P (2020). The healthiness and sustainability of national and global food based dietary guidelines: modelling study. BMJ.

[19] Food and Agriculture Organization of the United Nations (2014). The Second International Conference on Nutrition.

[20] Science Advice for Policy by European Academies (2020). A sustainable food system for the European Union. science advice for policy by European academies.

[21] Food and Agriculture Organization of the United Nations, Milan Urban Food Policy Pact, RUAF: The Milan Urban Food Policy Pact: Monitoring framework. Rome; 2019. http://www.fao.org/3/ca6144en/CA6144EN.pdf.

[22] United Nations (2021). The food systems summit: global dialogues.

[23] United Nations Development Program: The SDGs in action; nd. https://www.undp.org/sustainable-development-goals?utm_source=EN&utm_medium=GSR&utm_content=US_UNDP_PaidSearch_Brand_English&utm_campaign=CENTRAL&c_src=CENTRAL&c_src2=GSR&gclid=CjwKCAiAlfqOBhAeEiwAYi43F57wd59qGzk30p_6DgjgpG9rUB8l0AMEJvvt5Wrr2tmk9hTW-QscIxoCHaUQAvD_BwE

[24] Wood A, Halloran A, Gordon LJ (2020). Insight paper #2 of the Nordic food system transformation series: eight opportunities for Nordic collaboration on food system challenges.

[25] Bennett S, Agyepong IA, Sheikh K, Hanson K, Ssengooba F, Gilson L (2011). Building the field of health policy and systems research: an agenda for action. PLoS Med.

[26] Carlsson L, Callaghan E, Morley A, Broman G (2017). Food system sustainability across scales: a proposed local-to-global approach to community planning and assessment. Sustainability.

[27] Gilson L, Alliance for Health Policy and Systems Research, World Health Organization (2012). Health policy and systems research: a methodology reader.

[28] United Nations: The future is now. Science for achieving sustainable development. Global sustainable development report: Department of Economic and Social Affairs, United Nations; 2019. https://sustainabledevelopment.un.org/content/documents/24797GSDR_report_2019.pdf.

[29] Gilson L, Hanson K, Sheikh K, Agyepong IA, Ssengooba F, Bennett S (2011). Building the field of health policy and systems research: social science matters. PLoS Med.

[30] Broman GI, Robèrt KH (2017). A framework for strategic sustainable development. J Clean Prod.

[31] Carlsson L, Callaghan E, Broman G (2019). How can dietitians leverage change for sustainable food systems in Canada?. Can J Diet Pract Res.

[32] Food and Agriculture Organization of the United Nations: Sustainable food systems: Concept and framework; 2018. https://www.fao.org/3/ca2079en/CA2079EN.pdf.

[33] HLPE (2017). Nutrition and food systems.

[34] European Environment Agency (2017). Food in a green light– a systems approach for sustainable food.

[35] European Union (2018). Recipe for change. An agenda for a climate-smart and sustainable food system for a healthy Europe: report of the FOOD 2030 expert group.

[36] Patton MQ (2014). Qualitative research & evaluation methods: Integrating theory and practice.

[37] Guest G, Bunce A, Johnson L (2006). How many interviews are enough? An experiment with data saturation and variability. Field Methods.

[38] Kuzel AJ, Crabtrree BF, Miller WL (1999). Sampling in qualitative inquiry. Doing qualitative research.

[39] Boddy C (2012). The nominal group technique: an aid to brainstorming ideas in research. J Cetacean Res Manag.

[40] Harvey N, Holmes CA (2012). Nominal group technique: an effective method for obtaining group consensus. Int J Nurs Pract.

[41] Moore CM (1994). Group techniques for idea building.

[42] Brady PJ, Song HJ, Butler J (2017). Using an expert panel to develop social support program sequencing for young adults with type 1 diabetes. Health Promot Pract.

[43] Cerin E, Nathan A, Choi WK, Ngan W, Yin S, Thornton L, Barnett A (2019). Built and social environmental factors influencing healthy behaviours in older Chinese immigrants to Australia: a qualitative study. Int J Behav Nutr Phys Act.

[44] Boyatzis R (1998). Transforming qualitative information.

[45] Food and Agriculture Organization of the United Nations (2012). Sustainable diets and biodiversity – directions and solutions for policy, research and action.

[46] European Public Health Association (2017). Healthy and sustainable diets for European countries.

[47] Béné C, Oosterveer P, Lamotte L, Brouwer ID, de Haan S, Prager SD, Talsma EF, Khoury CK (2019). When food systems meet sustainability – current narratives and implications for actions. World Dev.

[48] European Comission (2020). Towards a sustainable food system.

[49] Ingram J, Fry P, Mathieu A (2010). Revealing different understandings of soil held by scientists and farmers in the context of soil protection and management. Land Use Policy.

[50] Tilman D, Clark M (2014). Global diets link environmental sustainability and human health. Nature.

[51] Popkin BM, Adair LS, Ng SW (2012). Global nutrition transition and the pandemic of obesity in developing countries. Nutr Rev.

[52] Paris JMG, Falkenberg T, Nöthlings U, Heinzel C, Borgemeister C, Escobar N (2021). Changing dietary patterns is necessary to improve the sustainability of Western diets from a One Health perspective. Sci Total Environ.

[53] Green R, Milner J, Dangour AD, Haines A, Chalabi Z, Markandya A, Spadaro J, Wilkinson P (2015). The potential to reduce greenhouse gas emissions in the UK through healthy and realistic dietary change. Clim Change.

[54] Poux X, Aubert P-M (2018). An agroecological Europe in 2050: multifunctional agriculture for healthy eating - findings from the Ten Years For Agroecology (TYFA) modelling exercise.

[55] Strapasson A, Woods J, Mbuk K. Land use futures in Europe. 2016. https://core.ac.uk/download/pdf/77010933.pdf.

[56] Rayner G, Barling D, Lang T (2008). Sustainable food systems in europe: policies, realities and futures. J Hunger Environ Nutr.

[57] Renzella J, Townsend N, Jewell J, Breda J, Roberts N, Rayner M, Wickramasinghe K (2018). What national and subnational interventions and policies based on Mediterranean and Nordic diets are recommended or implemented in the WHO European Region, and is there evidence of effectiveness in reducing noncommunicable diseases?.

[58] Sáez-Almendros S, Obrador B, Bach-Faig A, Serra-Majem L (2013). Environmental footprints of Mediterranean versus Western dietary patterns: beyond the health benefits of the Mediterranean diet. Environ Health.

[59] van Dooren C, Aiking H (2016). Defining a nutritionally healthy, environmentally friendly, and culturally acceptable Low Lands Diet. Int J Life Cycle Assess.

[60] McMichael AJ, Powles JW, Butler CD, Uauy R (2007). Food, livestock production, energy, climate change, and health. Lancet.

[61] Bechthold A, Boeing H, Tetens I, Schwingshackl L, Nöthlings U (2018). Perspective: food-based dietary guidelines in Europe-scientific concepts, current status, and perspectives. Adv Nutr.

[62] Pagliai G, Dinu M, Madarena MP, Bonaccio M, Iacoviello L, Sofi F (2021). Consumption of ultra-processed foods and health status: a systematic review and meta-analysis. Br J Nutr.

[63] Monteiro CA, Cannon G, Moubarac JC, Levy RB, Louzada MLC, Jaime PC (2018). The UN Decade of Nutrition, the NOVA food classification and the trouble with ultra-processing. Public Health Nutr.

[64] Romanos-Nanclares A, Toledo E, Sánchez-Bayona R, Sánchez-Quesada C, Martínez-González MÁ, Gea A. Healthful and unhealthful provegetarian food patterns and the incidence of breast cancer: results from a Mediterranean cohort. Nutrition. 2020;79–80:110884. 10.1016/j.nut.2020.110884 32736167

[65] Fardet A, Rock E (2020). Ultra-processed foods and food system sustainability: what are the links?. Sustainability.

[66] Alcorta A, Porta A, Tárrega A, Alvarez MD, Pilar Vaquero M (2021). Foods for plant-based diets: challenges and innovations. Foods.

[67] Bakaloudi DR, Halloran A, Rippin HL, Oikonomidou AC, Dardavesis TI, Williams J, Wickramasinghe K, Breda J, Chourdakis M (2021). Intake and adequacy of the vegan diet. A systematic review of the evidence. Clin Nutr.

[68] Macdiarmid JI, Kyle J, Horgan GW, Loe J, Fyfe C, Johnstone A, McNeill G (2012). Sustainable diets for the future: can we contribute to reducing greenhouse gas emissions by eating a healthy diet?. Am J Clin Nutr.

[69] Martini D, Tucci M, Bradfield J, Di Giorgio A, Marino M, Bo’ CD, Porrini M, Riso P (2021). Principles of sustainable healthy diets in worldwide dietary guidelines: efforts so far and future perspectives. Nutrients.

[70] Tetens I, Birt CA, Brink E, Bodenbach S, Bugel S, De Henauw S, Gronlund T, Julia C, Konde AB, Kromhout D (2020). Food-Based Dietary Guidelines-development of a conceptual framework for future Food-Based Dietary Guidelines in Europe: report of a Federation of European Nutrition Societies Task-Force Workshop in Copenhagen, 12–13 March 2018. Br J Nutr.

[71] Ernstoff A, Stylianou KS, Sahakian M, Godin L, Dauriat A, Humbert S, Erkman S, Jolliet O (2020). Towards win–win policies for healthy and sustainable diets in switzerland. Nutrients.

[72] Unicef (2021). Food-based dietary guidelines: a review of National Guidance for Children, Adolescents, and Women.

[73] Herforth A, Arimond M, Álvarez-Sánchez C, Coates J, Christianson K, Muehlhoff E (2019). A global review of food-based dietary guidelines. Adv Nutr.

[74] Garnett T, Mathewson S, Angelides P, Borthwick F, House C: Policies and actions to shift eating patterns: What works? A review of the evidence of the effectiveness of interventions aimed at shifting diets in more sustainable and healthy directions: University of Oxford; 2015. http://www.fcrn.org.uk/fcrn-publications/reports/policies-and-actions-shift-eatingpatterns-what-works.

[75] Ngqangashe Y, Goldman S, Schram A, Friel S (2022). A narrative review of regulatory governance factors that shape food and nutrition policies. Nutr Rev..

[76] World Health Organization (2014). Review of social determinants and the health divide in the WHO European Region.

[77] World Health Organization (2015). Using price policies to promote healthier diets.

[78] Andreyeva T, Marple K, Marinello S, Moore TE, Powell LM (2022). Outcomes following taxation of sugar-sweetened beverages. A systematic review and meta-analysis. JAMA Netw Open.

[79] Garnett T (2014). Changing what we eat A call for research & action on widespread adoption of sustainable healthy eating.

[80] Neto B (2020). Analysis of sustainability criteria from European public procurement schemes for foodservices. Sci Total Environ.

[81] European Union: Sustainable Public Meal Toolkit; nd. https://www.sustainable-public-meal.eu/en/.

[82] European Parliament (2014). Directive 2014/24/EU of the European Parliament and of the Council of 26 February 2014 on public procurement and repealing Directive 2004/18/EC Text with EEA 84 relevance.

[83] Morgan K (2008). Greening the realm: sustainable food chains and the public plate. Reg Stud.

[84] Nuffield Council on Bioethics (2007). Public health: ethical issues.

[85] Croker H, Packer J, Russell SJ, Stansfield C, Viner RM (2020). Front of pack nutritional labelling schemes: a systematic review and meta-analysis of recent evidence relating to objectively measured consumption and purchasing. J Hum Nutr Diet.

[86] El-Abbadi NH, Taylor SF, Micha R, Blumberg JB (2020). Nutrient profiling systems, front of pack labeling, and consumer behavior. Curr Atheroscler Rep.

[87] Kleef EV, Dagevos H (2015). The growing role of front-of-pack nutrition profile labeling: a consumer perspective on key issues and controversies. Crit Rev Food Sci Nutr.

[88] European Comission (2012). Policies to encourage sustainable consumption.

[89] Van Loo EJ, Hoefkens C, Verbeke W (2017). Healthy, sustainable and plant-based eating: perceived (mis)match and involvement-based consumer segments as targets for future policy. Food Policy.

[90] Hartmann C, Siegrist M (2017). Consumer perception and behaviour regarding sustainable protein consumption: a systematic review. Trends Food Sci Technol.

[91] Wyse R, Jackson JK, Delaney T, Grady A, Stacey F, Wolfenden L, Barnes C, McLaughlin M, Yoong SL (2021). The effectiveness of interventions delivered using digital food environments to encourage healthy food choices: a systematic review and meta-analysis. Nutrients.

[92] Halloran A, Faiz M, Chatterjee S, Clough I, Rippin H, Farrand C, Weerasinghe N, Flore R, Springhorn H, Breda J (2022). The cost of convenience: potential linkages between noncommunicable diseases and meal delivery apps. Lancet Reg Health Eur.

[93] United Nations Environment Programme (2021). Food waste index report 2021.

[94] WRAP (2015). The New Climate Economy: strategies to achieve economic and environmental gains by reducing food waste.

[95] Buttriss JL (2013). Food reformulation: the challenges to the food industry. Proc Nutr Soc.

[96] SCAR (2018). Assessment of research and innovation on food systems by European Member States: policy and funding analysis.

[97] World Health Organization (2018). Multisectoral and intersectoral action for improved health and well-being for all: mapping of the WHO European Region.

[98] Swedish Food Agency (2015). Find your way, to eat greener, not too much and be active.

[99] German Council for Sustainable Development (2013). The sustainable shopping basket. A guide to better shopping.

[100] Health Council of the Netherlands (2011). Guidelines for a healthy diet: the ecological perspective.

[101] Barling D (2011). The challenges facing contemporary food systems: European policy and governance pathways to sustainable food consumption and production. Agron Environ Soc.

[102] Committee on World Food Security (2017). Making a difference in food security and nutrition.

[103] Lambek N (2018). The UN committee on world food security’s break from the agricultural productivity trap. Transnatl Legal Theory.

[104] Wertheim-Heck SCO, Raneri JE (2020). Food policy and the unruliness of consumption: An intergenerational social practice approach to uncover transforming food consumption in modernizing Hanoi Vietnam. Glob Food Sec.

[105] Afshin A, Penalvo J, Del Gobbo L, Kashaf M, Micha R, Morrish K, Pearson-Stuttard J, Rehm C, Shangguan S, Smith JD (2015). CVD prevention through policy: a review of mass media, food/menu labeling, taxation/subsidies, built environment, school procurement, worksite wellness, and marketing standards to improve diet. Curr Cardiol Rep.

[106] De Lacy-Vawdon C, Livingstone C (2020). Defining the commercial determinants of health: a systematic review. BMC Public Health.

[107] Salm L, Nisbett N, Cramer L, Gillespie S, Thornton P (2021). How climate change interacts with inequity to affect nutrition. Wiley Interdiscip Rev Clim Change.

[108] World Health Organization (2021). Healthy and sustainable diets: report of an expert meeting on healthy and sustainable diets. A workshop to share challenges, identify knowledge gaps and receive feedback.

[109] Wood A, Halloran A, Gordon LJ (2020). Insight paper #1 of the Nordic food system transformation series: towards sustainable Nordic food systems – project overview.

