# Supplementary material for: Consensus-building around the conceptualisation and implementation of sustainable healthy diets: a foundation for policymakers
Source: BMC Public Health. 2022 Aug 4;22:1480. doi: 10.1186/s12889-022-13756-y (PMC9351147; doi:10.1186/s12889-022-13756-y)
Supplement: Supplementary file 1 — Additional file 1: Table S1. Script of questions asked during the semi-structured interview. [file 12889_2022_13756_MOESM1_ESM.pdf]

Table S1. Script of questions asked during the semi-structured interview.

Purpose of the questions	Questions
Background questions (Who?)	1. Currently, what is your job? What are you working on?<br>2. What are your areas of work/research expertise?<br>3. What experience do you have in the field of sustainability?<br>4. Have you been involved in any project or publication related to food sustainability?
Questions for conceptualizing healthy and sustainable diets (What?)	5. Defining a 'sustainable diet' is a complex challenge that requires consideration of multiple environmental, social, and economic factors. What is your opinion of the definition of sustainable diets provided by the FAO (2010) (displayed below)? What would you add or suggest for the purpose of explaining the concept?<br>Sustainable diets are those diets with low environmental impacts which contribute to food and nutrition security and to healthy life for present and future generations. Sustainable diets are protective and respectful of biodiversity and ecosystems, culturally acceptable, accessible, economically fair and affordable; nutritionally adequate, safe and healthy; while optimizing natural and human resources. FAO 2010.<br>6. Plant-based diets are proposed as a dietary solution. What is your opinion about meat- and dairy-free diets? Are they balanced, according to you? Do you think they are transferable to other cultures and contexts?<br>7. Which dietary patterns within the different cultural and geographical contexts of Europe could be a model for health and sustainability?<br>8. What changes are necessary to the current dietary patterns among the global population, taking into account all aspects related to food (type of product, packaging, etc.), to reduce environmental impact?
Questions about the actions and stakeholders needed to transform the food system into a healthy and sustainable system (How?)	9. In your opinion, what are the most appropriate tools for promoting sustainable healthy diets in our region? Please consider health and non-health sectors.<br>10. What aspects should be included in food-based dietary guidelines (FBDG) in relation to food sustainability, and what is our role in this process?<br>11. What kind of actions do you think would increase consumers' responsibility? In your experience, what are the main lines of action for promoting sustainable consumption habits among the population? List them in order of importance.<br>12. How do you consider promoting sustainable healthy diets?<br>13. What are the most appropriate tools for disseminating information

Purpose of the questions	Questions
	about sustainable consumption among the population? List them in order of relevance.<br>14. Currently, it is not straightforward for consumers to determine whether one food is more 'sustainable' than another. In your view, what is the best way to inform consumers?<br>15. In your opinion, what policies should be implemented to help consumers understand the environmental implications? Taking into account both high-income and low-income countries, what public policies do you think are necessary to reduce meat consumption in the world population?<br>16. What gaps between data and scientific evidence are there for relevant research into policies on sustainable healthy diets?<br>17. What facilitating elements and obstacles do you think there are for promoting a healthy and sustainable dietary pattern?<br>18. In current projects and other works related to the subject addressed, which important stakeholders are missing? Who else should we approach to join a consortium to make it more comprehensive?<br>19. What are your expectations of this project? In what ways do you think it will (or will not) contribute to improving current policies and practices with regards to sustainable diets?

This table provides all the questions that were asked during the semi-structured interview. The script consists of 19 questions, which are divided into 3 sections depending on the purpose: background questions, questions for conceptualizing healthy and sustainable diets, and questions about the actions and stakeholders needed to transform the food system into a healthy and sustainable system.
