# Supplementary material for: Consensus-building around the conceptualisation and implementation of sustainable healthy diets: a foundation for policymakers
Source: BMC Public Health. 2022 Aug 4;22:1480. doi: 10.1186/s12889-022-13756-y (PMC9351147; doi:10.1186/s12889-022-13756-y)
Supplement: Supplementary file 2 — Additional file 2: Table S2. Questions asked in the focus group session. [file 12889_2022_13756_MOESM2_ESM.pdf]

Table S2. Questions asked in the focus group session.

1. What specific actions should be taken to overcome the challenge?<br>2. What resources are needed to implement these actions?<br>3. Who are the relevant stakeholders that should be involved in action implementation?<br>4. Which important stakeholders are missing from this project and other relevant projects on the topic of healthy and sustainable dietary patterns?

This table provides the 4 questions that were asked during the focus group session. The questions were based on the actions recommended in the NGT groups, resources needed to implement those actions, and stakeholders that should be involved.
