# Supplementary material for: Consensus-building around the conceptualisation and implementation of sustainable healthy diets: a foundation for policymakers
Source: BMC Public Health. 2022 Aug 4;22:1480. doi: 10.1186/s12889-022-13756-y (PMC9351147; doi:10.1186/s12889-022-13756-y)
Supplement: Supplementary file 3 — Additional file 3: Table S3. Number of participants in each focus group, by professional profile. [file 12889_2022_13756_MOESM3_ESM.pdf]

Table S3. Number of participants in each focus group, by professional profile.

	Group 1. Supply	Group 2. Demand	Group 3. Public Procurement	Group 4. Food and Packaging Waste/Food Production (Technology, Water Crisis) and Reformulation	TOTAL
Environmental Footprints	2	3	0	2	7
Communication and Policies	1	2	2	2	7
Food Profiling - Prioritization and Modelling	3	1	1	1	6
General Health View (Health, Research, Policies)	1	2	1	0	4
Government Perspective	1	1	2	0	4

This table provides more detailed information about the number of participants in each focus group, taking into account their professional profile (Environmental Footprints, Food Profiling - Prioritization and Modelling, General Health View (Health, Research, Policies), Communication and Policies, and Government Perspective.
