# Supplementary material for: Consensus-building around the conceptualisation and implementation of sustainable healthy diets: a foundation for policymakers
Source: BMC Public Health. 2022 Aug 4;22:1480. doi: 10.1186/s12889-022-13756-y (PMC9351147; doi:10.1186/s12889-022-13756-y)
Supplement: Supplementary file 4 — Additional file 4: List of members of the DPHS project Expert Group. [file 12889_2022_13756_MOESM4_ESM.pdf]

List of members of the DPHS project Expert Group:

Betina Bergmann, Isabel Castanheira, Michail Chourdakis, Nathalie Farpour-Lambert, Mads Frederik Fischer-Møller, Ujué Fresán, Lene Frost, Alessandro Galli, Trine Grønlund, Mirjana Gurninovic, Fatima Hachem, Trine Hahnemann, Bent Egberg Mikkelsen, Else Molander, Pedro Moreira, Patrícia Padrão, Nuno Queiroz, Mike Rayner, Ana Rito, Sirpa Sarlio, Elena Smirnova, Peter Sousa, Marco Springmann, Elisabeth Temme, Corné van Dooren, Pieter Van't veer, Jan Wollgast, Amanda Wood, María José Yusta.
